# Leading by Example: Web-Based Sexual Health Influencers Among Men Who Have Sex With Men Have Higher HIV and Syphilis Testing Rates in China

**DOI:** 10.2196/10171

**Published:** 2019-01-21

**Authors:** Dan Wu, Weiming Tang, Haidong Lu, Tiange P Zhang, Bolin Cao, Jason J Ong, Amy Lee, Chuncheng Liu, Wenting Huang, Rong Fu, Katherine Li, Stephen W Pan, Ye Zhang, Hongyun Fu, Chongyi Wei, Joseph D Tucker

**Affiliations:** 1 University North Carolina at Chapel Hill, Project-China Guangzhou China; 2 Social Entrepreneurship to Spur Health Global Guangzhou China; 3 Dermatology Hospital of Southern Medical University Guangzhou China; 4 Faculty of Infectious and Tropical Diseases London School of Hygiene and Tropical Medicine London United Kingdom; 5 Department of Epidemiology University of North Carolina at Chapel Hill Gillings School of Global Public Health Chapel Hill, NC United States; 6 Stritch School of Medicine Loyola University Chicago Maywood, IL United States; 7 School of Media and Communication Shenzhen University Shenzhen China; 8 Central Clinical School Monash University Monash Australia; 9 Department of Sociology University of California San Diego, CA United States; 10 Weill Cornell Medical College New York, NY United States; 11 Department of Health and Environmental Sciences Xi’an Jiaotong-Liverpool University Suzhou China; 12 Kirby Institute University of New South Wales Sydney Australia; 13 Eastern Virginia Medical School Norfolk, VA United States; 14 Department of Social and Behavior Health Sciences Rutgers School of Public Health Piscataway, NJ United States

**Keywords:** health promotion, peer influence, internet, social networks, social media, HIV, syphilis, men who have sex with men, China

## Abstract

**Background:**

The spread of healthy behaviors through social networks may be accelerated by influential individuals. Previous studies have used lay health influencers to prevent sexually transmitted infections (STIs) among internet-using men who have sex with men (MSM). However, there is a lack of understanding of the characteristics of this key subset of MSM.

**Objective:**

This study aimed to examine sociodemographic characteristics, HIV and syphilis testing, and sexual behaviors of Web-based MSM sexual health influencers (SHIs) in China, defined as individuals with relatively stronger influence on spreading HIV and STI information online.

**Methods:**

A Web-based survey of MSM was conducted in August 2017 as a final follow-up of a randomized controlled trial promoting HIV testing in 8 Chinese cities. Men were recruited through a gay social networking mobile phone app and were included if they were born biologically male, aged 16 years and above, ever had sex with another man, and HIV negative or with unknown HIV status. Information regarding sociodemographic characteristics, sexual behaviors, and HIV and syphilis testing was obtained. We assessed men’s Web-based sexual health influence using a standardized 6-item opinion leadership scale focused on HIV and STI information. Influencers were defined as those whose mean score ranked within the top 13% (a higher score means greater influence). We used multivariable linear and logistic regression models to measure Web-based sexual health influence’s association with HIV and syphilis testing, controlling for intervention trial effects, age, education, income, and marital status.

**Results:**

Overall, 1031 men completed the survey. Most men were younger than 30 years (819/1031, 79.43%) and had at least college education (667/1031, 64.69%). Influencers were more likely to get tested for HIV (73/132, 55.3% vs 337/899, 37.5%; *P*<.001) and syphilis (35/132, 26.5% vs 137/899, 15.2%; *P*=.001) in the last 3 months compared with noninfluencers. There were no significant differences in condomless sex with male partners (26/132, 19.7% vs 203/899, 22.6%; *P*=.46), mean number of male sex partners (1.32 vs 1.11; *P*=.16) in the last 3 months, and mainly meeting male sex partners online in the last 12 months (97/132, 73.5% vs 669/899, 74.4%; *P*=.82) between influencers and noninfluencers. Regression analyses showed that influencers had higher odds of HIV testing (adjusted odds ratio, AOR 2.16, 95% CI 1.48-3.17) and syphilis testing (AOR 1.99, 95% CI 1.28-3.10) in the last 3 months.

**Conclusions:**

We identified Web-based SHIs who might be more likely to help promote healthy HIV and syphilis testing behaviors through MSM populations. Leveraging existing influencers may help improve HIV and syphilis testing among their networks.

## Introduction

### Background

Men who have sex with men (MSM) continue to be disproportionately affected by HIV globally [1]. In Asia, MSM account for 18% of all newly identified HIV diagnosis, and the rising prevalence has been documented in China [2-6]. The national HIV prevalence among MSM in China in 2015 was 8.0%, 200 times higher than 0.04% in China’s general population [7]. The low HIV testing rate continues to contribute to the spread of HIV among MSM. Although China has significantly expanded its efforts to control HIV transmission [8], systematic reviews suggest that the HIV testing rate among the Chinese MSM remains low [9-11]. Barriers to HIV testing include the lack of MSM community engagement, hesitancy to access facility-based services, and low level of trust toward facility-based services [12].

Influential individuals facilitate the spread of certain behaviors within a population, as demonstrated in both diffusion research [13,14] and social network research [15-17]. The social diffusion theory suggests that behavior change in a population can be initiated and diffused to others if a behavior is visibly endorsed by enough natural and influential opinion leaders within the population [14]. These influential individuals informed the concept of *popular opinion leaders (POLs)* and the development of POL-based HIV interventions that successfully identified and trained popular individuals to spread HIV prevention messages to peers [18,19]. Social networks can amplify the spread of behavior through interpersonal ties and have been used by public health interventions to promote a range of health behaviors [20-22]. The targeting of influential individuals also makes social network interventions more effective and efficient, possibly because of optimal properties associated with an influential individual’s network structure [17]. This body of evidence indicates that behavioral change in social networks may be accelerated through influential individuals.

In the internet era, MSM increasingly turn to Web-based networks and social media to look for sexual health information [23-25]. Popular MSM who frequently share sexual health information with their friends or social media followers may influence the health behaviors of people among their networks. However, understanding of such influential individuals in Web-based MSM networks remains incomplete. First, although social media is more available to MSM as a way to disseminate health knowledge [26], its use among Web-based influential individuals has not been fully explored [19]. Second, unlike POL interventions where the behaviors spread by influential individuals are purposefully established by POL-targeted training, Web-based networks also facilitate the spread of behaviors that are naturally endorsed by influential individuals themselves [15]. However, the sexual risk behaviors, HIV and syphilis testing behaviors of Web-based influential individuals have not been thoroughly described, creating uncertainty to the degree of these individuals’ positive influence.

Knowledge of both the use of social media and the set of health behaviors endorsed by Web-based influential individuals can inform the development of MSM-led, network-based interventions. The high rates of internet and mobile phone usage in China particularly provide a strong foundation for network-based interventions on social media [27-29].

### Objectives

In this study, we aimed to examine *Web-based sexual health influencers (SHIs)*, defined as individuals with strong influence on spreading HIV and sexually transmitted infection (STI) information online, among Chinese MSM to describe their social-behavioral characteristics, including sexual behaviors, HIV and syphilis testing, social media engagement, community engagement, and HIV-relevant psychological profiles such as anticipated HIV stigma and HIV testing self-efficacy.

## Methods

### Study Population

An online survey of 1031 MSM was conducted in August 2017 as a final follow-up of a step-wedged randomized controlled trial to improve HIV testing rates in 8 Chinese cities (Guangzhou, Jiangmen, Zhuhai, and Shenzhen in Guangdong province and Yantai, Jinan, Qingdao, and Jining in Shandong province). The cities of each province were randomly assigned the order of intervention and paired into 4 groups accordingly. The methods have been described in detail elsewhere [30]. Men were recruited through a gay social networking mobile phone app, Blued, by sending a survey invitation to registered users in the 8 selected cities. Men were included if they were born biologically male, aged 16 years and above, ever had sex with another man, and HIV negative or with unknown HIV status. All individuals completed informed consent. The intervention included individual- and community-level components. We sent campaign images and texts promoting HIV testing, along with local testing site information, to all participants privately on WeChat, an instant messaging tool (individual-level component). Local community-based organizations organized a crowdsourcing contest soliciting individual stories relevant to HIV testing among the MSM community with an aim of improving community engagement (community-level component). We followed the participants quarterly for 1 year. We conducted the final follow-up survey immediately after the completion of the final round of intervention. We conducted a secondary analysis of the final follow-up survey data for this study.

### Measures

#### Sociodemographic, Sexual History, and Intervention Exposure Measurement

We collected information about men’s sociodemographic characteristics: age, residence status (nonmigrants or migrants), marital status (never married, currently married, divorced or widowed), educational attainment (high school or below, some college, and college or above), and annual income (US $<2500, US $2501-8500, US $8501-14,000, and US $>14,000). Sexual history included their sexual orientation (gay, bisexual, or unsure), sexual orientation disclosure to others (yes or no), sexual orientation disclosure to health care providers (yes or no), number of male sex partners in the last 3 months, whether had regular and/or casual male partners in the last 3 months (yes or no), and whether met male sex partner online, including website and social media platforms (yes or no). Exposure to interventional materials, including images, texts, local testing sites information, and a local crowdsourcing contest promoting HIV testing, was also noted (yes or no).

#### Web-Based Sexual Health Influence Measurement

Personal influence, or communication between the communicator and receiver, has been noted historically as a powerful factor in explaining and predicting people’s behavior [31]. It has been conceptualized as opinion leadership in studies of the diffusion process, while this diffusion leads to people’s adoption of a new idea, behavior, or product [14]. We adapted the scale from a standardized 6-item opinion leadership scale [31] to measure *Web-based SHI* (abbreviated in text as *influencers*). A similar 6-item scale had been used to assess opinion leadership among a Web-based Taiwanese MSM population [19]. The scale assessed men’s influence on spreading HIV and STI information online. The 6 items included: (1) how often they talked to their MSM friends or followers about HIV and STI, (2) how much information they provided when talking about HIV and STI, (3) how many MSM they told about HIV and STI, (4) how likely they were to be asked for more information about HIV and STI, (5) who communicated more information about HIV and STI: the participants or their MSM friends or followers, or almost equal, and (6) how often they were used as a source of advice. All items were assessed on a 5-point Likert scale. The Cronbach alpha of the scale is .937 in this study. On the basis of Rogers’ diffusion of innovation theory, approximately 15% of the individuals of a community are early adopters of an innovation and can consequently influence others as well as shape social norms [14]. We defined *Web-based SHIs* as those whose opinion leadership mean score ranked within the top 15% (mean score >3) in the cohort (mean score ranged from 1 to 5). However, because of difficulty with multiple observations showing the same score at the 15% cut-off, a slightly tighter but most close cut-off 13% was chosen, rather than 15%.

#### Behavioral and Social Media Engagement Measurement

Men were asked about any condomless sex with male partners in the last 3 months (yes or no), HIV testing in the last 3 months including either facility-based testing or self-testing (yes or no), and syphilis testing in the last 3 months (yes or no). We asked about their social media engagement, defined as whether they reported using Weibo (microblog similar to Twitter), WeChat, and QQ (both are instant messaging mobile apps) or Blued (a gay dating app) in the last 3 months to give or receive information about HIV testing, except for the information delivered by the trial (yes or no). We asked men to report the number of followers on their various social media platforms, including Weibo, WeChat, QQ, and Blued. We also asked the respondents to self-report approximately how many of their MSM friends or followers on social media have gone for an HIV test after their intervention, using a 5-point Likert scale question (none at all, a few, some, many, or quite a lot).

#### Community Engagement and HIV-Relevant Psychological Measurement

We measured community engagement, anticipated HIV stigma [32], HIV testing social norms [33], and HIV testing self-efficacy [34]. Community engagement was measured by 6 items, with each using binary responses (yes or no), which assessed men’s level of engagement in sexual health activities. Score of community engagement ranged from 0 to 6, and a higher score means better community engagement in sexual health. The 7-item anticipated HIV stigma scale asked participants about their own feelings about themselves if they had HIV as well as perceived discriminating attitudes from other people. HIV testing social norms were measured using a validated 6-item scale asking about men’s opinions of the gay community’s attitudes toward HIV testing. HIV testing self-efficacy was measured using a 6-item scale. Answers to items of these scales were given in a 4-point Likert format: strongly disagree=1, disagree=2, agree=3, and strongly agree=4. Mean scores for anticipated HIV stigma, HIV testing social norms, and self-efficacy were calculated by averaging the summed values of all items, ranging from 1 to 4. A higher score means a higher level of anticipated stigma, better perceived social norms, or better self-efficacy. All scales have been used and evaluated in China before. In this study, the Cronbach alpha values for community engagement, anticipated HIV stigma, HIV testing social norms, and HIV testing self-efficacy were .709, .880, .494, and .787, respectively (see Multimedia Appendix 1 for more detailed responses to individual items of the scales).

### Statistical Analysis

Due to the small cells of self-reported influence on others’ adoption of HIV test within influencers, we dichotomized the variable and grouped *many* and *quite a lot* into 1 category for further analysis. Descriptive analysis was conducted to describe the differences in sociodemographic characteristics, sexual behaviors, HIV and syphilis testing history, exposure to interventional materials, use of social media platforms, anticipated HIV-related stigma, HIV testing social norms, HIV testing self-efficacy, and community engagement between influencers and noninfluencers. Chi-square or Fisher exact test was used for categorical variables, and independent sample *t* test was used for continuous variables.

We conducted multivariable linear or logistic regression analyses to examine the association between opinion leadership and various characteristics outcomes aforementioned. Mean differences in continuous variables between influencers and noninfluencers were evaluated, whereas odds ratios were reported for binary outcomes. Given the effects of trial interventions on these outcomes as well as its potential association with leadership, we controlled all previous interventions as predictors in the crude model (model 1). In addition, sociodemographic factors including age, education, income, and marital status were controlled in an extended model (model 2). We reported 95% CIs and *P* values. A *P* value of less than .05 was considered statistically significant. Data were analyzed with SAS software, version 9.4 (SAS Institute, Cary, USA).

### Ethical Statement

Ethical approval was obtained from the institutional review committees at the Dermatology Hospital of Southern Medical University (Guangzhou, China), Shandong University (Jinan, China), University of North Carolina at Chapel Hill (Chapel Hill, North Carolina), and the University of California, San Francisco (San Francisco, California) before the launch of the survey.

## Results

### Sociodemographic and Behavioral Characteristics

Among 1031 men, 132 (132/1031, 12.80%) had a mean leadership score of greater than 3 and were categorized as influencers and 899 (899/1031, 87.20%) were noninfluencers (Multimedia Appendix 1). Most men were younger than 30 years (819/1031, 79.44%), were living in the sampling city (926/1031, 89.82%), were never married (907/1031, 87.97%), obtained college or above education (667/1031, 64.69%), and self-identified as gay (741/1031, 71.87%; see Table 1). Compared with noninfluencers, influencers were more likely to disclose their sexual orientation to others (100/132, 75.8% vs 580/899, 64.5%; *P*=.01), disclose sexual orientation to health care providers (37/132, 28.0% vs 178/899, 19.8%; *P*=.03), have a casual male partner in the last 3 months (63/132, 47.7% vs 315/899, 35.0%; *P*=.05), test for HIV in the last 3 months (73/132, 55.3% vs 337/899, 37.5%; *P*<.001), test for syphilis in the last 3 months (35/132, 26.5% vs 137/899, 15.2%; *P*=.001), and self-report that many or quite a lot of others had taken an HIV test after their intervention within their network (42/132, 31.8% vs 38/899, 4.2%; *P*<.001). There were no significant differences between the 2 groups in terms of the average number of male sex partners (1.32 vs 1.11; *P*=.16), condomless sex with male partners in the last 3 months (26/132, 19.7% vs 203/899, 22.6%; *P*=.46), and mainly meeting male sex partners online in the last 12 months (97/132, 73.5% vs 669/899, 74.4%; *P*=.82).

### Social Media Engagement and Participation in Program Intervention

Table 2 shows exposure to the intervention trial and social media engagement by influencers and noninfluencers. Being in the same cohort, influencers were more likely to have seen our interventional materials, including images promoting HIV testing (125/132, 94.7% vs 759/899, 84.4%; *P*=.002), texts promoting HIV testing (120/132, 90.9% vs 672/899, 74.7%; *P*<.001), and a local crowdsourcing contest (63/132, 47.7% vs 312/899, 34.7%; *P*=.004). In terms of using social media platforms to give and receive any extra information relevant to HIV testing (except for the information delivered by the trial), influencers were more likely to give or receive extra information than noninfluencers on Weibo (42/132, 31.8% vs 112/899, 12.5%; *P*<.001), WeChat (85/132, 64.4% vs 275/899, 30.6%; *P*<.001), QQ (54/132, 40.9% vs 125/899, 13.9%; *P*<.001%), and mobile apps (42/132, 31.8% vs 169/899, 18.8% *P*=.001). As shown in Table 3, compared with noninfluencers, influencers had significantly more QQ followers (238 vs 159; *P*=.03), less Blued followers (172 vs 466; *P*=.003), a lower anticipated HIV stigma score (2.7 vs 2.9; *P*<.001), a higher HIV testing self-efficacy score (3.4 vs 3.1; *P*<.001), and a higher community engagement score (4.0 vs 2.5; *P*<.001).

### Factors Related to Being a Sexual Health Influencer

As shown in Table 4, being influencers was associated with lower mean scores of anticipated HIV stigma (mean difference −0.22; *P*=.02), higher mean scores of HIV testing self-efficacy (mean difference 0.24; *P*=.01), and higher summed scores of community engagement (mean difference 1.48; *P*<.001). The differences remained significant after controlling for sociodemographic factors additionally in model 2.

**Table 1:** Demographic characteristics of Web-based sexual health influencers and noninfluencers in a men who have sex with men cohort in China in 2016 to 2017.

Demographic characteristics		Total (N=1031)	Noninfluencers (N=899)	Influencers (N=132)	*P* value
**Age (years), n (%)**					.41
	<20	174 (16.88)	154 (17.1)	20 (15.2)	
	20-29	645 (62.56)	555 (61.7)	90 (68.2)	
	30-39	170 (16.49)	154 (17.1)	16 (12.1)	
	≥40	42 (4.07)	36 (4.0)	6 (4.5)	
**Residence** **status, n (%)**					.27
	Nonmigrants	926 (89.82)	811 (90.2)	115 (87.1)	
	Migrants	105 (10.18)	88 (9.8)	17 (12.9)	
**Marital status, n (%)**					.96
	Never married	907 (87.97)	790 (87.9)	117 (88.6)	
	Currently married	89 (8.63)	78 (8.7)	11 (8.3)	
	Divorced or widowed	35 (3.40)	31 (3.4)	4 (3.0)	
**Educational level attained, n (%)**					.17
	High school or below	364 (35.31)	327 (36.4)	37 (28.0)	
	Some college	285 (27.64)	245 (27.3)	40 (30.3)	
	College or above	382 (37.05)	327 (36.4)	55 (41.7)	
**Annual income (US $), n (%)**					.17
	≤2500	235 (22.79)	202 (22.5)	33 (25)	
	2501-8500	544 (52.76)	474 (52.7)	70 (53.0)	
	8501-14,000	159 (15.42)	146 (16.2)	13 (9.8)	
	>14,000	93 (9.02)	77 (8.6)	16 (12.1)	
**Sexual orientation, n (%)**					.86
	Homosexual	741 (71.87)	647 (72.0)	94 (71.2)	
	Bisexual	252 (24.44)	218 (24.2)	34 (25.8)	
	Unsure	38 (3.69)	34 (3.8)	4 (3.0)	
Disclose sexual orientation to anyone^a^, n (%)		680 (65.96)	580 (64.5)	100 (75.8)	.01
Disclose sexual orientation to health providers^a^, n (%)		215 (20.85)	178 (19.8)	37 (28.0)	.03
Number of male sex partners in the last 3 months, mean (SD)		1.14 (1.5)	1.11 (1.5)	1.32 (1.8)	.16
Mainly met male sex partners online in the last 12 months^a,b^, n (%)		766 (74.30)	669 (74.4)	97 (73.5)	.82
Had a regular male partner in the last 3 months^a,c^, n (%)		351 (34.04)	307 (34.1)	44 (33.3)	.85
Had a casual male partner in the last 3 months^a,d^, n (%)		378 (36.66)	315 (35.0)	63 (47.7)	.005
Condomless sex with male partners in the last 3 months^a^, n (%)		229 (22.21)	203 (22.6)	26 (19.7)	.46
HIV test in the last 3 months^a,e^, n (%)		410 (39.77)	337 (37.5)	73 (55.3)	<.001
Syphilis test in the last 3 months^a^, n (%)		172 (16.68)	137 (15.2)	35 (26.5)	.001
Self-reported influence on others’ adoption of an HIV test after their Web-based intervention (Many or quite a lot), n (%)		80 (7.76)	38 (4.2)	42 (31.8)	<.001

^a^The response was yes for these variables.

^b^Mainly met with male sexual partners through a website or social media platforms.

^c^Regular male partner was defined as the one who was in a stable relationship (over 3 months) that did not involve transactional sex.

^d^Casual male partner was defined as a male sexual partner that the participant did not consider to be his regular partner.

^e^Either facility-based testing or self-testing.

**Table 2:** Exposure to the trial intervention reported by Web-based sexual health influencers and noninfluencers in a men who have sex with men cohort in China in 2016 to 2017.

Binary outcomes		Total (N=1031)	Noninfluencers (N=899)	Influencers (N=132)	*P* value
**Exposure to the trial intervention materials, n (%)**					
	Have seen any images promoting HIV testing	884 (85.74)	759 (84.4)	125 (94.7)	.002
	Have seen any texts promoting HIV testing	792 (76.82)	672 (74.7)	120 (90.9)	<.001
	Have seen local testing sites information	864 (83.80)	749 (83.3)	115 (87.1)	.27
	Have seen a local crowdsourcing contest	375 (36.37)	312 (34.7)	63 (47.7)	.004
**Give or receive anything related to HIV testing on social media platforms except for the information from the trial, n (%)**					
	Using Weibo to give or receive information	154 (14.94)	112 (12.5)	42 (31.8)	<.001
	Using WeChat to give or receive information	360 (34.92)	275 (30.6)	85 (64.4)	<.001
	Using QQ to give or receive information	179 (17.36)	125 (13.9)	54 (40.9)	<.001
	Using Blued to give or receive information	211 (20.47)	169 (18.8)	42 (31.8)	.001

**Table 3:** Number of social media followers, HIV-relevant psychological profiles, and community engagement by Web-based sexual health influencers and noninfluencers in a men who have sex with men cohort in China in 2016 to 2017 (N=1031).

Continuous outcomes	Noninfluencers	Influencers	*P* value
Number of Weibo followers, mean (SD)	269 (1430)	740 (5267)	.31
Number of WeChat followers, mean (SD)	168 (317)	749 (4725)	.17
Number of QQ followers, mean (SD)	159 (334)	238 (374)	.03
Number of Blued followers, mean (SD)	466 (2709)	172 (418)	.003
Anticipated HIV stigma^a^, mean score (SD)	2.9 (0.7)	2.7 (0.8)	<.001
HIV testing social norms^b^, mean score (SD)	2.9 (0.4)	2.8 (0.4)	.76
HIV testing self-efficacy^b^, mean score (SD)	3.1 (0.5)	3.4 (0.5)	<.001
Community engagement^c^, mean score (SD)	2.5 (1.7)	4.0 (1.6)	<.001

^a^Mean scores of anticipated HIV stigma ranged from 1 to 4, and a higher score means a higher level of anticipated stigma.

^b^Mean scores of HIV testing social norms and self-efficacy ranged from 1 to 4, and higher mean scores mean better perceived social norms and better self-efficacy.

^c^Score of community engagement ranged from 0 to 6, and a higher score means better community engagement in sexual health.

**Table 4:** Association between Web-based sexual health influence and continuous outcomes in a men who have sex with men cohort in China in 2016 to 2017 (N=1031).

Continuous outcomes^a^	Model 1^b^		Model 2^c^	
	Estimated mean difference (95% CI)	*P* value	Estimated mean difference (95% CI)	*P* value
Anticipated HIV stigma	−0.22 (−0.40 to −0.05)	.02	−0.23 (−0.35 to −0.12)	<.001
HIV testing social norms	−0.01 (−0.11 to 0.08)	.77	−0.02 (−0.10 to 0.06)	.69
HIV testing self-efficacy	0.24 (0.07 to 0.41)	.01	0.25 (0.16 to 0.34)	<.001
Community engagement	1.48 (1.06 to 1.90)	<.001	1.50 (1.19 to 1.81)	<.001

^a^Reference group is nonsexual health influencers.

^b^Model 1 was only adjusted for a previous intervention package to promote HIV testing among the cohort.

^c^Model 2 was additionally adjusted for age, education, income, and marital status.

**Table 5:** Association between Web-based sexual health influence and binary outcomes in a men who have sex with men cohort in China in 2016 to 2017 (N=1031).

Behavioral outcomes^a^		Model 1^b^		Model 2^c^	
		Estimated odds ratio (95% CI)	*P* value	Estimated odds ratio (95% CI)	*P* value
**Testing behaviors and condom use in the last 3 months**					
	Overall HIV testing	2.12 (1.45-3.09)	<.001	2.16 (1.48-3.17)	<.001
	HIV self-testing	1.62 (1.09-2.42)	.02	1.64 (1.10-2.46)	.02
	HIV facility-based testing	2.59 (1.75-3.82)	<.001	2.66 (1.79-3.96)	<.001
	Consistent condom use	1.29 (0.78-2.12)	.32	1.33 (0.80-2.19)	.27
	Syphilis testing	1.94 (1.26-3.01)	<.01	1.99 (1.28-3.10)	<.01
**Social media engagement^d^**					
	Using Weibo to give or receive information	1.88 (1.20-2.97)	<.001	1.90 (1.19-3.02)	<.01
	Using WeChat to give or receive information	3.56 (1.78-7.13)	<.001	3.79 (1.87-7.66)	<.001
	Using QQ to give or receive information	2.76 (1.71-4.45)	<.001	2.91 (1.79-4.75)	<.001
	Using an app to give or receive information	1.07 (0.68-1.69)	.76	1.04 (0.66-1.64)	.87
**Self-reported influence on others’ HIV test uptake**					
	Many or quite a lot of people took a test	6.81 (4.14-11.2)	<.001	7.62 (4.55-12.78)	<.001

^a^Reference group is nonsexual health influencers.

^b^Model 1 was only adjusted for a previous intervention package to promote HIV testing among the cohort.

^c^Model 2 was additionally adjusted for age, education, income, and marital status.

^d^Social media engagement was defined as whether they reported using Weibo, WeChat, QQ, or a mobile app in the last 3 months to give or receive information about HIV testing, except for the information delivered by the trial.

Table 5 shows comparisons of binary outcomes between the 2 groups. Being influencers was found to be significantly associated with higher odds of HIV testing in the last 3 months (adjusted odds ratio, AOR 2.16, 95% CI 1.48-3.17), HIV self-testing in the last 3 months (AOR 1.64, 95% CI 1.10-2.46), HIV facility-based testing in the last 3 months (AOR 2.66, 95% CI 1.79-3.96), and syphilis testing in the last 3 months (AOR 1.95, 95% CI 1.25-3.03). Influencers were 1.90 (95% CI 1.19-3.02), 3.79 (95% CI 1.87-7.66), and 2.91 (95% CI 1.79-4.75) times more likely to use Weibo, WeChat, and QQ, respectively to give or receive extra HIV testing–relevant information than noninfluencers. In terms of self-reported influence on others’ HIV test uptake, influencers were 7.62 (95% CI 4.55-12.78) times more likely to report that many or quite a lot of people within their network have gone for an HIV test after their intervention. However, opinion leadership was found not to be associated with consistent condom use.

## Discussion

### Principal Findings

Influential individuals may help promote health behaviors within their networks; however, there is insufficient understanding of the characteristics of Web-based influential individuals who are increasingly important to health promotion in the internet era. By examining social media engagement and health behaviors of Web-based SHIs among Chinese MSM, this study extends the literature by illuminating the degree to which existing influential individuals may be useful agents in the Web-based virtual space and by informing the development of network-based social media interventions. Our study found that influencers had higher social media engagement for HIV testing, higher likelihood of HIV and syphilis testing, and did not have increased sexual risk behavior. This group could become key leaders within network-based social media MSM HIV interventions.

We found that influencers had a higher HIV testing rate than noninfluencers. The HIV testing rate of influencers was higher than that of Chinese MSM in another postintervention study [35]. After adjusting for the intervention, influencers in this study also had a higher likelihood of HIV testing. Their higher HIV testing may be related to increased community engagement in sexual health [36] and HIV testing–related social media use [37]. The higher rates of HIV testing among influencers may also be related to lower HIV stigma and higher testing self-efficacy, which are 2 important contributors to test uptake [32,38]. Influencers also had a higher rate of syphilis testing than noninfluencers in the last 3 months (26.5% vs 15.2%). This is consistent with previous studies that found community engagement in sexual health to be associated with increased syphilis testing [36]. This suggests that influencers could be helpful in promoting dual HIV and syphilis testing, given that these related infections often co-occur among MSM in China [39].

In terms of influence on others’ adoption of HIV test, we found influencers were significantly associated with reporting that many people within their Web-based social network have taken an HIV test after their intervention. This may be explained by greater social media engagement and larger social network sizes among influencers. Influencers had a greater exposure to our trial intervention materials, including seeing any images or texts promoting HIV testing and local testing sites information. They were also more active in using various social media platforms to communicate with others about HIV testing. Being more active in receiving and disseminating sexual health information indicated that natural influencers may be more central to information flow, hence facilitating healthy behavior spread within their social network.

Finally, there was no significant difference between influencers and noninfluencers with regard to sexual behaviors. Specifically, rates for condomless sex, having a regular male partner, meeting sex partners online, and the mean number of male sex partners were similar. These findings are relevant to the growing body of research on internet use and sexual risk behaviors among MSM. On the one hand, internet use and social media are believed to be an avenue for meeting MSM, who then engage in risky behaviors associated with transmission of HIV and other STIs [40,41]. However, our study revealed that influencers had less followers than noninfluencers in Blued, the most popular gay social networking app in China. This indicates that influencers may in fact use this app less often for finding dates online. Furthermore, social media use could also potentially decrease sexual risk behaviors as it allows MSM to discuss with others about sexual health and learn about HIV and STI prevention [42]. Influencers in this study had a higher degree of online usage and communication with other MSM; however, they do not have increased sexual risk behaviors.

### Limitations

We noted some limitations of the study. First, the study tended to focus on describing the influencers’ characteristics and behaviors. Yet, descriptive studies on influencers are valuable, given that health behaviors are known to spread from person to person in social networks [43,44]. Our study may also provide a mechanism (ie, centrality in information flow) explaining why naturally existent influencers may facilitate behavioral change within their own network. Second, we only evaluated opinion leadership in the final follow-up survey and were not able to examine the longitudinal effects of the intervention on the relationship between opinion leadership and testing behaviors. Instead, we controlled for the intervention in the models to eliminate the confounding effects of interventions on the outcomes. Third, we used self-reported data to measure leadership and their influence on others’ HIV test uptake. More reliable measurement and more robust research are necessary to evaluate the effect of influencers within MSM social networks with regard to behavior change. Incorporating social network measurement (eg, eigenvector centrality) and personal influence (eg, opinion leadership) and measuring their effects on positive behavior spread are worth consideration. Finally, our cross-sectional design makes it difficult to determine causal relationships.

### Implications

Our findings have implications for strengthening HIV and syphilis testing interventions. Web-based SHIs could be useful in a range of testing promotion models, particularly network-based social media approaches. Vulnerable populations such as MSM may distrust outside authorities but find advice from known influencers who carry credibility [45]. For example, influencers could serve as steering committee members of crowdsourcing contests that aim to promote testing [30,46]. Influencers could also serve as leaders in network-based social media interventions to allow health messages to reach more MSM [37]. We found a higher rate of social media engagement about HIV testing and a higher likelihood of having used social media for HIV testing in influencers compared with other MSM, suggesting influencers may be readily incorporated into social media HIV interventions [37]. Influencers additionally contribute by being well positioned in social networks to spread behavior change. Interventions that operate through existing influencers’ social networks hold promise for reaching vulnerable communities, particularly when formal prevention infrastructure supports are limited [17,47].

## Appendix

Multimedia Appendix 1 Detailed responses to individual items of scales used in the study.

## Abbreviations

AOR: adjusted odds ratio
MSM: men who have sex with men
POL: popular opinion leader
SHI: sexual health influencer
STI: sexually transmitted infection
