# Supplementary material for: Leading by Example: Web-Based Sexual Health Influencers Among Men Who Have Sex With Men Have Higher HIV and Syphilis Testing Rates in China
Source: J Med Internet Res. 2019 Jan 21;21(1):e10171. doi: 10.2196/10171 (PMC6360381; doi:10.2196/10171)
Supplement: Multimedia Appendix 1 [file jmir_v21i1e10171_app1.pdf]

Table 1: Responses to online leadership scale and classification of online sexual health influencers in a men who have sex with men (MSM) online cohort in China in 2016-2017 (n=1031)

		Frequency	Percent
**The online opinion leadership scale**			
In general, how often do you talk to your MSM friends/followers about HIV/STI online?	Never	314	30.5
	Rarely	418	40.5
	Sometimes	231	22.4
	Often	39	3.8
	Very often	29	2.8
When you talk to your MSM friends/followers about HIV/STI online, how much information do you provide them?	Very little	352	34.1
	Small amount	256	24.8
	A moderate amount	273	26.5
	Large amount	104	10.1
	A great deal	46	4.5
During the past 3 months, how many MSM have you told about HIV/STI online?	No one	411	39.9
	A small number	343	33.3
	A moderate number	198	19.2
	A large number	45	4.4
	A great number	34	3.3
Compared with your circle of MSM friends, how likely are you to be asked for more information about HIV/STI online?	Very unlikely	454	44.0
	Unlikely	328	31.8
	Neutral	180	17.5
	Likely	41	4.0
	Very likely	28	2.7
In a discussion of HIV/STI online, which of the following happens most often online?	Your MSM friends/followers tell you about HIV/STI	395	38.3
	Your MSM friends/followers tell you more than you tell them about HIV/STI	200	19.4
	You and your MSM friends/followers tell each other almost equally	290	28.1
	You tell your MSM friends/followers more than they tell you about HIV/STI	93	9.0
	You tell your MSM friends/followers about HIV/STI	53	5.1
Overall in all of your discussions with MSM friends/followers online, how often are you used as a source of advice?	Never	411	39.9
	Rarely	305	29.6
	Sometimes	235	22.8
	Often	62	6.0
	Very often	18	1.7
The Cronbach's Alpha of the scale is 0.937, suggesting excellent internal consistency reliability.			
**Classification of influencers and non-influencers**			

Influencers[*]	Mean score > 3	132	12.8
Non-influencers	Mean score =<3	899	87.2

[*]Sexual health influencers.

Table 2: Responses to community engagement scale by Chinese MSM in 2016-2017 (n=1031)

	Yes		No	
	Frequency	Percent	Frequency	Percent
Are you aware of any ongoing community events promoting HIV testing among MSM?	505	49.0	526	51.0
Have you ever volunteered at a health clinic or other location that provided sexual health services among MSM?	169	16.4	862	83.6
Have you ever helped organize a testing and/or awareness campaign (e.g. HIV, condom use, etc.) that promoted sexual health among MSM?	271	26.3	760	73.7
Have you ever encouraged someone else to get tested for HIV and/or another sexually transmitted disease?	732	71.0	299	29.0
Have you ever accompanied a friend or partner to a testing facility to get tested for HIV and/or another sexually transmitted disease?	463	44.9	568	55.1
Have you ever participated in online forums or discussions on social media (i.e., WeChat, Weibo, or other on-line communities) about HIV/STI testing or related services?	648	62.9	383	37.1
Cronbach's Alpha 0.709				

Table 3: Responses to anticipated stigma scale by Chinese MSM in 2016-2017 (n=1031)

	Strongly agree		Agree		Disagree		Strongly disagree	
	Frequency	Percent	Frequency	Percent	Frequency	Percent	Frequency	Percent
If I had HIV, I'd worry about people discriminating against me.	411	39.9	403	39.1	159	15.4	58	5.6
If I got infected with HIV no one would date or become involved with me.	338	32.8	414	40.2	214	20.8	65	6.3
If I got infected with HIV, no one would want to have sex with me.	426	41.3	381	37.0	178	17.3	46	4.5
If I got infected with HIV, I would work hard to keep my HIV status a secret.	323	31.3	425	41.2	213	20.7	70	6.8
Upon learning I contracted HIV, I would feel set apart and isolated from the rest of the world.	283	27.4	376	36.5	293	28.4	79	7.7
If I got infected with HIV, I would feel I was not as good a person as others.	229	22.2	305	29.6	354	34.3	143	13.9
I would never feel ashamed of getting HIV*.	160	15.5	359	34.8	383	37.1	129	12.5
Cronbach's Alpha: 0.880								

*Reversely coded.

**Table 4: Responses to HIV testing social norms scale by Chinese MSM in 2016-2017 (n=1031)X**	Strongly agree		Agree		Disagree		Strongly disagree	
	Frequency	Percent	Frequency	Percent	Frequency	Percent	Frequency	Percent
Most gay men who want to get tested but are afraid to get tested.	245	23.8	427	41.4	298	28.9	61	5.9
Most gay men who get tested do not want others to find out they were tested.	346	33.6	437	42.4	206	20.0	42	4.1
Most gay men want to get tested for HIV.	326	31.6	489	47.4	200	19.4	16	1.6
Most gay men who want to get tested will tell their partners they want to get tested.	192	18.6	486	47.1	311	30.2	42	4.1
Most gay men have been tested for HIV.	144	14.0	329	31.9	480	46.6	78	7.6
Most gay men get tested for HIV only if they are sick or feel uncomfortable.	215	20.9	499	48.4	260	25.2	57	5.5
Cronbach's Alpha: 0.494								

Table 5: Responses to HIV testing self-efficacy scale by Chinese MSM in 2016-2017 (n=1031)

	Strongly agree		Agree		Disagree		Strongly disagree	
	Frequency	Percent	Frequency	Percent	Frequency	Percent	Frequency	Percent
You would feel comfortable discussing HIV testing with a potential partner.	266	25.8	528	51.2	209	20.3	28	2.7
You feel confident that you could refuse to have sex with a partner who did not want to undergo HIV testing.	375	36.4	449	43.5	167	16.2	40	3.9
You feel confident that you could persuade your partner to undergo HIV testing.	286	27.7	619	60.0	116	11.3	10	1.0
You can have an HIV test if you wish.	508	49.3	493	47.8	20	1.9	10	1.0
You will have an HIV test even if you are afraid to know the results.	442	42.9	528	51.2	47	4.6	14	1.4
You have confidence that you will undergo HIV testing regularly.	186	18.0	458	44.4	361	35.0	26	2.5
Cronbach's Alpha 0.787								
